# Supratentorial clear cell meningioma in a child: A rare tumor at unusual location

**DOI:** 10.4103/1817-1745.76114

**Published:** 2010

**Authors:** Rakesh Ranjan, Shrividya Sethuraman

**Affiliations:** Department of Neurosurgery, Aditya Birla Memorial Hospital, Pune, India; 1 Department of Pathology, Aditya Birla Memorial Hospital, Pune, India

**Keywords:** Clear cell meningioma, pediatric brain tumor, pediatric meningioma, supratentorial meningioma

## Abstract

Clear cell meningioma is a rare subtype of meningioma seen mainly in pediatric patients. Supratentorial location is an unusual site of occurrence and its natural history and prognosis are not well described in the literature. We present a unique case of a left-sided frontoparietal tumor in a 15-year-old child who was managed successfully with gross total surgical excision and is recurrence-free at 18 months follow-up. A favorable clinical behavior and a longer symptom-free interval can be expected after gross total removal. The patients should be followed after successful surgery and other modalities of therapy should be used for recurrence.

## Introduction

Meningiomas occurring in the first two decades of life are uncommon, accounting for only about 1-2% of all brain tumors.[1,2] Clear cell meningioma (CCM) is a rare phenotype, with a potentially aggressive clinical course and risk of recurrence.[3–5] The occurrence of this tumor in the supratentorial space is rare and adds to the uncertainty about its clinical course and prognosis. We report a case of such tumor at this unusual location and discuss about the dilemmas associated with the management and long-term prognosis.

## Case Report

**Figure 1:**
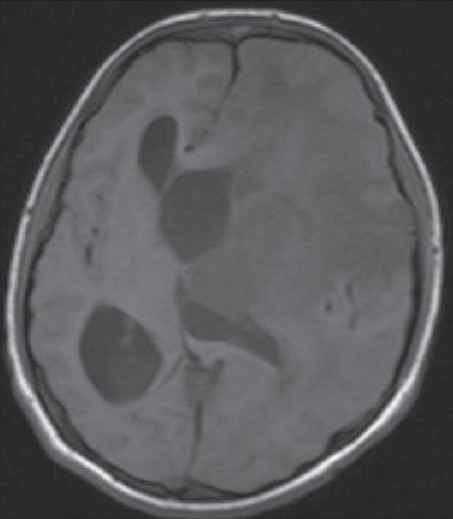
Axial section of the T1-weighted magnetic resonance imaging image of the brain showing the tumor with a midline shift

**Figure 2:**
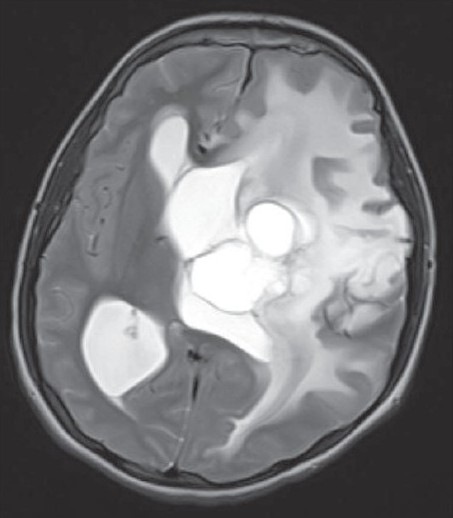
Axial section of the T2-weighted magnetic resonance imaging image of the brain showing solid and cystic components and peritumoral edema

**Figure 3:**
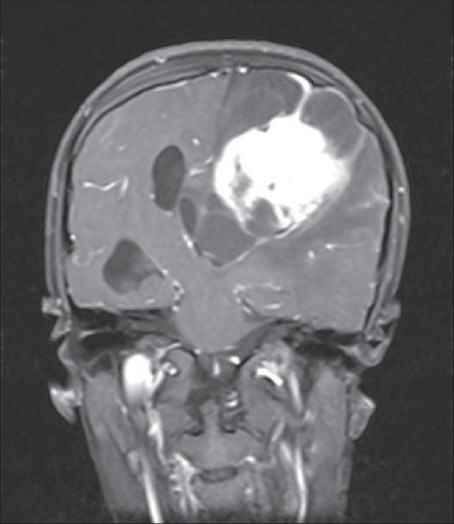
Coronal section of the contrast-enhanced magnetic resonance imaging image showing heterogenous enhancement of the tumor

**Figure 4:**
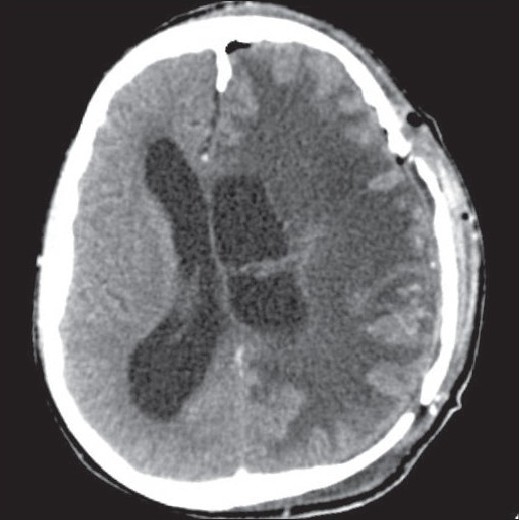
Postoperative contrast-enhanced computed tomography scan image showing no residual tumor

**Figure 5:**
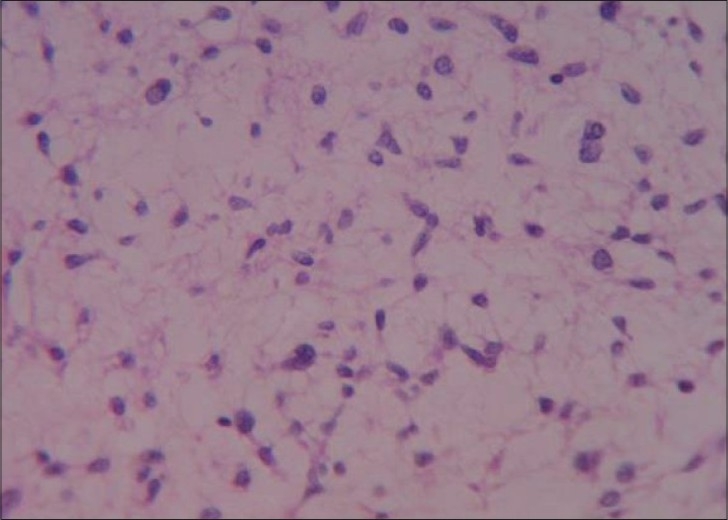
Clear cell meningioma composed of loosely arranged cells in a myxoid stroma. A typical intranuclear inclusion is seen on the upper left

**Figure 6:**
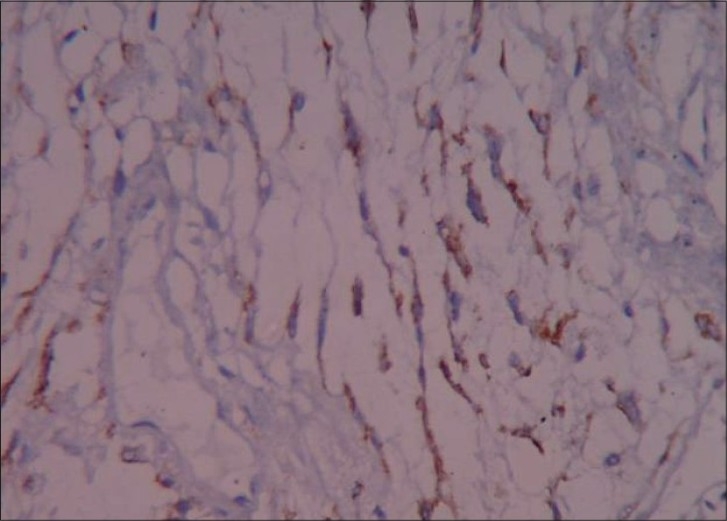
Clear cell meningioma with strong EMA immunoreactivity in the tumor cells

A 15-year-old male child presented with history of worsening right-sided weakness of 2 years duration. He had progressive vision loss in the left eye for the past 1 year. He had multiple episodes of focal-onset generalized tonic clonic seizures with poor drug compliance. Headache with projectile vomitings had started lately. On clinical examination, the patient was blind in the left eye, with a normal right eye. There was mild right-sided seventh nerve weakness and hemiparesis. A magnetic resonance imaging scan of the brain revealed a left-sided large supratentorial tumor extending from the brain surface in the frontal and parietal cortex to the atrium and frontal horn of the lateral ventricle, causing significant compression [Figure 1]. There was brain edema with evidence of tentorial herniation. The tumor had both solid and cystic components [Figure 2]. The tumor showed heterogenous contrast enhancement after gadolinium injection [Figure 3]. Taking into consideration the radiological findings, a possibility of primitive neuroectodermal tumor was made. At surgery, after the bone flap was elevated, the cystic component of the tumor was found to be infiltrating the duramater near the lateral parietal region, causing an impression over the inner surface of the bone. The cystic component of the tumor contained xanthochromic fluid. The solid component was firm, moderately vascular and poorly suckable. A gross total resection of the tumor was performed along with resection of the infiltrated duramater overlying the parietal lobe. The patient had complete relief of headache and vomiting in the postoperative period and was free of seizures on phenytoin. A postoperative computed tomography (contrast) scan of the brain [Figure 4] revealed total excision of the tumor. Histopathology of the tumor revealed a diagnosis of CCM WHO grade II. The tumor was composed of lobules of loosely arranged cells set in a very loose myxoid stroma. The cells had scanty cytoplasm with indistinct cell margins and slender ovoid nuclei, some with intranuclear cytoplasmic inclusions [Figure 5]. The tumor cells were positive for Epithelial membrane antigen (EMA) [Figure 6] and vimentin and negative for S-100, synaptophysin, Glial fibrillary acidic protien (GFAP), cytokeratin and desmin. The MIB -1 index of the tumor was 8%. The patient is doing well at 18 months of follow-up.

## Discussion

CCM is a recently described histological subtype of meningioma and is reported to occur more frequently in the pediatric age group.[3,5–8] The most common site of occurrences are the spinal canal (intradural, 48%), the cerebellopontine angle, tentorium, skull base and foramen magnum.[1,4,5]

Supratentorial location of pediatric CCM is rare. In a review of 35 cases of intracranial CCM, Ma *et al*. reported only two pediatric patients as having supratentorial intraparenchymal CCM.[5,6] They treated the patients with intracranial and spinal radiation after surgical removal.[8]

Radiologically, the lesions show strong and homogenous enhancement following contrast injection.[5] The histopathological differential diagnoses include microcystic meningioma, hemangioblastoma and clear cell ependymoma.[6] The characteristic histology and immunohistochemistry leads to the confirmation of diagnosis.[9–11] The clinical behavior of these tumors has been shown to be aggressive, with a high incidence of local recurrence, cerebrospinal fluid (CSF) spread or metastases.[3,5–7] However, the rate of increase of dimension of such a tumor is not known. There is no definitive method to identify the potential of recurrence of this tumor. In a study comparing the levels of proliferating cell nuclear antigen (pCNA) and MIB-1 index in nonrecurring and recurring CCM, it was reported that the mean values were 10.4% and 11% for nonrecurring and recurring tumors, respectively, for pCNA.[9] The mean values for the MIB-1 index were 7.4% and 13.3% for the nonrecurring and recurring tumors, respectively. Furthermore, no close association was noted between the recurrence of tumor and factors such as mitotic activity, pCNA proliferation indices, percent S-phase determination or DNA ploidy status.[11] The long-term outcome of CCM is unknown, with only few cases free of recurrence at 5 years or more in the follow-up period.[11] Local recurrence of the tumor has been reported even with low levels of MIB-1 index. The treatment strategies have been mainly reported for spinal CCMs. Gross total resection has been considered to be the treatment of choice and radiosurgery and chemotherapy have been considered treatment options in recurring cases.[1,4]

This child presented with a lesion in the left frontoparietal region. Such a location of the tumor with absence of any subarachnoid or CSF seedling is extremely rare. The radiological study revealed the tumor to be heterogenous in consistency with solid and cystic components and had a nonuniform enhancement after gadolinium injection. At surgery of this child, it was revealed that the cyst had caused an impression on the inner surface of the parietal bone, which may be suggestive of the gradual progression of the tumor. With reference to the literature review, CCM may have benign histological characteristics, but, clinically, it shows a high risk of progression and recurrence. Given the large dimension of the tumor (6-7 cm), local impression on the bone and long duration of symptoms, the rate of growth of the tumor seems to be slow in this case. The MIB-1 index in the patient was 8%, making the course of illness unpredictable. The recurrence rate for these tumors has mainly been reported with reference to spinal CCM and the guidelines for therapy are based there upon. Given the behavior of the tumor in the present case, we can safely recommend a close follow-up in the postoperative period, with radiotherapy reserved for cases with recurrence.
